# Profile of hepatitis B virus resistance mutations against nucleoside/nucleotide analogue treatment in Chinese patients with chronic hepatitis B

**DOI:** 10.1186/1743-422X-10-313

**Published:** 2013-10-25

**Authors:** Jun Lei, Ying Wang, Li-Li Wang, Shao-Jun Zhang, Wei Chen, Zhi-Gang Bai, Lv-Ye Xu

**Affiliations:** 1 Department of Infectious Diseases, Wuhan Neurologist Hospital and General Hospital of The Yangtze River Shipping, Wuhan, China

**Keywords:** Hepatitis B virus, Reverse transcriptase, Resistance, Virological breakthrough, Nucleos(t)ide analogues

## Abstract

**Aim:**

Antiviral drug-resistant HBV mutants are complex and currently partly understood. This study was performed to analyze the profile of hepatitis B virus (HBV) resistance mutations against nucleos(t)ide analogues (NAs) in patients with chronic hepatitis B (CHB).

**Methods:**

This was a population-based cross-sectional study. Serum samples of 179 patients with virological breakthrough undergoing different NAs treatment were obtained between January 2008 and December 2012. The HBV reverse transcriptase region was sequenced and the following NAs-resistant changes including rtL80, rtI169, rtV173, rtL180, rtA181, rtT184, rtA194, rtS202, rtM204, rtN236 and rtM250 were analyzed.

**Results:**

In this cohort, 21.2% (38/179) were genotypes B and 78.8% (141/179) were genotypes C; and 89.4% (160/179) of them detected NAs-resistant mutations. The prevalence of HBV mutations at rtM204 was 93.0% (106/114) in patients with lamivudine (LAM) or telbivudine (LdT)-based therapies, and that of rtN236 mutations was 76.1% (35/46) in patients with adefovir dipivoxil (ADV)-based therapies. Among LAM/LdT based therapies, HBV rtM204I was significantly associated with HBV rtL80I/V mutations [rtM204I+rtL80I/V (50.0%, 32/64) vs. rtM204V+rtL80I/V (27.3%,9/33), *P*=0.032]; while the HBV rtM204V mutations was significantly associated with HBV rtL180M mutations [rtM204V+rtL180M (100%, 33/33) vs. rtM204I+rtL180M (60.9%, 39/64), *P*<0.001]. Additionally, HBV rtA181 mutations were observed in 19.3% (22/114) of patients with LAM/LdT-based therapy and 23.9% (11/46) of patients with ADV-based therapy.

**Conclusions:**

Majority of virological breakthrough is associated with NAs-resistant HBV, and the mutation patterns of NAs-resistant HBV are complicated in real clinical practice.

## Introduction

For the ability to inhibit the activity of hepatitis B virus (HBV) reverse transcriptase (RT), nucleos(t)ide analogues (NAs) such as lamivudine (LAM), adefovir dipivoxil (ADV), entecavir (ETV) and telbivudine (LdT) are approved for the treatment of chronic hepatitis B (CHB) in China [1,2]. However, with wide-scale and long-term use of NAs against HBV, more attention has been focused on the risk of development of NAs resistance; and many evidences indicate that genotypic mutations at specific amino acid positions in the HBV RT region would reduce its susceptibility to NAs and HBV resistance, which is now recognized as the single most important factor of virological breakthrough and failure treatment of NAs [3-5].

At present, many mutations in the RT gene of HBV have been reported to be associated with classical antiviral resistance, and mutations of rtA181, rtM204 and rtN236 have been reported to be associated with major primary drug resistance and are responsible for reduced treatment susceptibility resulting from antiviral agents [6]. However, primary drug resistances are inseparable from secondary and/or compensatory mutations such as rtV173, rtL180 and rt250, which could restore the RT activity defects of HBV caused by primary drug resistance [6-9]. According to previously studies, the patterns of genotypic resistance in the HBV polymerase can be categorized into five specific evolutionary pathways [10,11], including L-nucleoside pathway (rtM204I [or V or I/V]), the acyclic phosphonate pathway (rtN236T), the shared pathway (rtA181T [or V or T/V]) of both L-nucleoside and acyclic phosphonate, ETV resistance pathway (rtL180M+rtM204V with one of either rtT184, S202 or M250 residue changes) and multidrug resistance pathways (rtA181T+rtI233V+rtN236T+rtM250L).

However, the profile data of classical antiviral resistance mutations belonging to different evolutionary pathways is limited in real clinical practice. Thus, this study is designed to characterize the molecular features of nucleos(t)ide analogues resistance (NAr) mutation positions belonging to different NAr pathways within HBV RT sequences.

## Methods

### Study design and patients

This was a population-based cross-sectional study. A total of 1308 CHB patients undergoing LAM, ADV, ETV or LdT treatment in single, sequential or combination between January 2008 and December 2012 were screened, and only 179 patients with virological breakthrough were finally included and analyzed in present study. The inclusion criteria were CHB patients received NAs treatment for more than 6 months and had virological breakthrough with HBV DNA ≥ 1.0×10^4^ copies/mL. The exclusion criteria were patients with hepatitis C virus (HCV) or human immunodeficiency virus (HIV) coinfection, autoimmune liver disease and alcohol or drug abuse. This study was also approved by the Ethics Committee of Wuhan Neurologist Hospital and General Hospital of The Yangtze River Shipping in accordance with the Declaration of Helsinki.

### Laboratory tests

Serum HBV DNA was quantified by fluorescence quantitative polymerase chain reaction (PCR) (Da’an Gene, Guangzhou, China) with the lowest detection limit of 1,000 copies/mL. HBV gene fragment (nt54-1278) encompassing the complete RT gene was amplified by PCR. Sequencing was performed using the ABI 3730xl DNA Analyzer(Hitachi, Tokyo, Japan). Sequencing data were analyzed using the Vector NTI Suite software package (Invitrogen, California, USA). HBV genotypes assignment was based on the phylogenetic analysis of the 1225-bp-long S/P-gene sequence (nt 54–1278) of HBV genome. Mutations at 12 locations (including rtL80, rtI169, rt173, rt180, rt181, rtT184, rtA194, rt202, rt204, rtI233, rt236, and rt250) in the RT gene belonging to different NAr pathways were analyzed using DNAStar version 5.0 (DNAStar, Madison, WI, USA) software, and all above HBV NAs-resistant associated mutations were detected when virological breakthrough occurred.

### Definition

The clinical diagnosis of CHB was made according to guideline on prevention and treatment of chronic hepatitis B in China [12,13]. Virological breakthrough was defined as having HBV DNA increase by >1 log from nadir level or a re-detection of HBV DNA at levels at least 10-fold higher than the lower limit of detection of the assay after having an undetectable result [14].

### Statistical analysis

Quantitative values expressed as mean plus standard deviation or range, and qualitative values were were presented as number and percent. Group comparisons between qualitative values were performed using Chi-square tests using SPSS 17.0 software (SPSS Inc., Chicago, IL, United States). P value of less than 0.05 was considered statistically significant.

## Results

In this cohort, there were 63 (35.2%) females and 116 (64.8%) males with an average age of 38 years (range 21–63), and the HBeAg positivity rate was 67.6% (121/179). Among those 179 HBV patients, 21.2% (38/179) and 78.8% (141/179) were infected with HBV genotypes B and C, respectively; 89.4% (160/179) of the patients detected NAs-resistant mutations including 114 patients with LAM/LdT-based therapies and 46 patients with ADV-based therapies. Among the 160 patients with NAs-resistant mutations, up to 7 antiviral strategies were involved which manifested as monotherapy [LAM (n=72), ADV (n=41) or LdT (n=13)], LAM plus ADV combination therapy (n=11), LAM switch-to ADV (n=18), or ETV sequential therapies (n=5).

In present study, NAs associated mutations were detected at positions rtL80, rtV173, rtL180, rtA181, rtS202, rtM204, rtN236 and rtM250, but not at positions rtI169, rtT184, rtA194 or rtI233. Among LAM/LdT-based treatment (n=114), the prevalence rates of rtM204 mutations were 100% (85/85), 77.8% (14/18), 50.0% (4/8) and 100% (3/3) in patients receiving LAM or LdT monotherapy, ADV switch-to, ADV add-on, or ETV switch-to therapy respectively. And among those rtM204 mutations, rtM204I, rtM204V and rtM204I/V accounted for 60.4% (64/106), 31.1% (33/106) and 8.5% (9/106), respectively. Thus, our finding further confirms that rtM204 should be considered as an important primary LAM/LdT-resistance mutation position in real clinical practice, and rtM204I mutation is the most common type.

Among those rtM204 mutations, a total of 14 types of rtM204 containing mutant patterns were observed, and rtM204I/V+rtL180M (78/106, 73.6%) and rtM204 I/V+rtL80I/V (48/106, 45.3%) were the two most common mutation patterns (Table 1). Compared with the rtM204V mutants, the rtM204I mutants were preferentially accompanied by the rtL80I/V mutations with a statistically significant difference [rtM204I+rtL80I/V (50.0%, 32/64) *vs.* rtM204V+rtL80I/V (27.3%, 9/33), *P*=0.032]. By contrast, all rtM204V mutants were found to be coexistent with rtL180M mutations, which was significantly higher than that of rtM204I mutants [rtM204V+rtL180M (100%, 33/33) *vs.* rtM204I+rtL180M (60.9%, 39/64), *P*<0.001].

**Table 1:** The patterns of HBV rtM204 mutations in this cohort

**Types of mutation patterns**	**Percentage**
Combination mutation patterns including rtM204I	64
rtM204I+ rtL180M	26(40.6%)
rtM204I+ rtL80I/V	16(25.0%%)
rtM204I+ rtL80I/V+ rtL180M	7(10.9%)
rtM204I+ rtL80I/V+ rtA181T + rtM250L	5(7.8%)
rtM204I+ rtL180M + rtV173M	6(9.4%)
rtM204I+ rtL80I/V+ rtA181T	4(6.3%)
Combination mutation patterns including rtM204V	33
rtM204V+ rtL180M	15(45.5)
rtM204V+ rtV173L + rtL180M	9(27.3)
rtM204V+ rtL80I/V+ rtL180M	5(15.2)
rtM204V+ rtL80I/V + rtV173L + rtL180M	4(12.1)
Combination mutation patterns including rtM204I/V	9
rtM204I/V+ rtL80I/V++ rtA181T	3(33.3%)
rtM204I/V+ rtL180M+ rtA181T	2(22.2%)
rtM204I/V+ rtL80I/V+ rtL180M	3(33.3%)
rtM204I/V+ rtL80I/V+ rtV173L + rtL180M	1(11.1%)

Among ADV-based treatment (n=46), the rtN236T mutations were detected in 78% (32/41), 66.7% (2/3) and 50% (1/2) of patients receiving ADV monotherapy, LAM add-on, or ETV switch-to therapies, respectively. Additionally, among LAM/LdT-based monotherapy, 50% of patients who (4/8) received ADV add-on also detected rtN236T mutations.

Recently, HBV rtA181T/V mutations had been widely concerned and it was regarded to be a multidrug resistance which not only could result in resistance to ADV but also reduce sensitivity to LAM and LdT. In this cohort, HBV rtA181T/V mutation was observed in 19.3% (22/114) of patients with LAM/LdT-based therapies and 23.9% (11/46) of patients with ADV-based therapies. It was worth mentioning that this mutation was detected in all 11 patients who received LAM plus ADV combination therapy (8 from LAM/LdT-based combination therapy and 3 from ADV-based combination therapy). Our findings suggested that rtA181T/V mutations were common among patients with NAs resistance, and it should be regularly monitored in real clinical practice.

## Discussion

In this study, classical antiviral resistance mutations within the HBV RT region, which belonged to diverse evolutionary pathways, were analyzed among 179 CHB patients with virological breakthrough. Among those patients, 89.4% (160/179) detected antiviral-resistant HBV mutations, which indicated that the emergency of resistance was the main reason for virological breakthrough and failure of treatment. In this cohort, three classic evolutionary pathways of L-nucleoside pathway against LAM, LdT or ETV (rtM204 I/V), the acyclic phosphonate pathway (rtN236T) against ADV and the shared pathway against both LAM and ADV (rtA181T T/V) were all involved, and our findings were also supported by recent studies [15,16]. Because tenofovir disoproxil fumarate was currently unavailable in China, its associated resistance mutations were not reported in this study.

Recently, rtI233 mutation has been reported to be associated with primary poor or failure response to ADV [17]; however, this mutation was not observed in present study. So we inferred that HBV rtI233 mutation was not a common strain in real life of ADV-experienced patients and its clinical significance needs to be studied in future. It has reported that rtA194T mutation was associated with tenofovir (TDF), and it could be selected by long-term application of ADV [18]. However, this mutation was also not detected. Thus, we also thought that HBV rtA194T might be not a common mutation induced by ADV and TDF would be used as a rescue medication for ADV treatment failure.

In this study, majority of patients had resistance that was associated with the L-nucleoside pathway [rtM204I (or V or I/V)], which could potentially lead to resistance to LAM, ETV and LdT treatment in LAM-experienced patients. The highest prevalence rate of genotypic resistance to the L-nucleoside pathway was in agreement with the largest percentage (66.3%, 106/160) of LAM-based patients in our cohort. Indeed, this high prevalence of rtM204 mutation was caused by the long-term use of LAM in China. So we speculated the presence of rtM204 mutation carriers might constitute an unavoidable challenge for the effective control of antiviral-resistant HBV in the future because it is difficult to clear such resistant viral strains [19].

Although preliminary data using ADV belonging to acyclic phosphonates on LAM-resistance patients seem promising, a shared pathway (rtA181T/V) of both L-nucleosides and acyclic phosphonates could be selected, which would affect both LAM and ADV sensitivity [20]. In present study, HBV rtA181T/V mutation was observed in 19.3% (22/114) of patients with LAM/LdT-based therapy and 23.9% (11/46) of patients with ADV-based therapy, and our results suggested that rtA181T/V antiviral resistance mutation has become a relatively common phenomenon in Chinese patients. Thus, the potential risk of the spread of such resistant viral strains (HBV rtM204 or rtA181mutants) is of therapeutic concern and represents a crucial public health threat [11,20].

The most obvious limitation of the study is its limited sample size, thus clinical trials with large sample sizes were required to reveal the comprehensive and accurate profile of HBV resistance mutations against NAs.

## Conclusions

Our findings indicate that there is a complex pool of resistant HBV in real life of CHB patients with virological breakthrough. Thus, monitoring HBV genotypic resistance and shared resistant pathway pertaining to antiviral agents would help to optimize or rescue current antiviral therapies and avoid the outbreak of clinical deterioration.

## Competing interests

The contents are solely the responsibility of the authors.

## Authors’ contributions

XLY was involved in the study concept and design, statistical analysis and interpretation of the data, drafting and critical revision of the manuscript. LJ was involved in the study concept and design, acquisition of the data, statistical analysis and interpretation of the data, drafting and critical revision of the manuscript and study supervision. WLL was involved in statistical analysis and interpretation of the data and critical revision of the manuscript. ZSJ, CW and BZJ were involved in the study concept and design, and in the critical revision of the manuscript. All authors have read and approved the final manuscript.
